# Intelligence Process vs. Content and Academic Performance: A Trip through a House of Mirrors

**DOI:** 10.3390/jintelligence10040128

**Published:** 2022-12-19

**Authors:** Phillip L. Ackerman

**Affiliations:** School of Psychology, Georgia Institute of Technology, 654 Cherry Street, Atlanta, GA 30332, USA; plackerman@gatech.edu; Tel.: +1-404-894-5611

**Keywords:** academic performance, Binet, Wechsler, Spearman, validity

## Abstract

The main purpose of modern intelligence tests has been to predict individual differences in academic performance, first of children, then adolescents, and later extending to adults. From the earliest Binet–Simon scales to current times, most one-on-one omnibus intelligence assessments include both process subtests (e.g., memory, reasoning) and content subtests (e.g., vocabulary, information). As somewhat parallel developments, intelligence theorists have argued about the primacy of the process components or the content components reflecting intelligence, with many modern researchers proposing that process constructs like working memory are the fundamental determinant of individual differences in intelligence. To address whether there is an adequate basis for re-configuring intelligence assessments from content or mixed content and process measures to all-process measures, the question to be answered in this paper is whether intellectual process assessments are more or less valid predictors of academic success, in comparison to content measures. A brief review of the history of intelligence assessment is provided with respect to these issues, and a number of problems and limitations of process measures is discussed. In the final analysis, there is insufficient justification for using process-only measures to the exclusion of content measures, and the limited data available point to the idea that content-dominated measures are more highly predictive of academic success than are process measures.

## 1. Overview

The goal for this paper was simple in the planning. Given that various theories, research programs, and intelligence/ability tests differ in their emphasis on the ‘processes’ underlying intelligence and the ‘content’ of intelligence, it seemed a relatively straightforward plan to review the existing measures and their relative validities for predicting academic success; an aim that *should* have been easy to carry out, for example, by reviewing various articles and extending the search to perhaps include validity data from test manuals. Instead, as I progressed with this project, I found myself in a house of mirrors, with far too little substance, and too many reflections in the shadows. I realized that in order to answer the main question, there were additional, more fundamental issues that had to be addressed, and further (to anticipate the conclusion of this paper), the answer to whether process or content assessments provide greater predictive power for criterion validity is not known, even with nearly 120 years since the introduction of the first modern intelligence scales.

## 2. Process vs. Content

Starting an article contrasting two constructs should be a relatively simple task of defining the constructs. In this case, the task is a bit more difficult. Perhaps the most straightforward way to envision these two constructs is to cite Guilford (1967), in the context of his Structure of Intellect model. Guilford identified “content” as one of the three major dimensions of his model, with four different contents: Figural, Symbolic, Semantic, and Behavioral. A second dimension, called “operations”, corresponds to a set of processes, including: Evaluation, Convergent Production, Divergent Production, Memory, and Cognition. (The third dimension, ‘products’, refers to “the way or form in which any information occurs,” and includes Units, Classes, Relations, Systems, Transformations, and Implications (p. 63).) From this perspective, it should be clear that in the context of assessment, operations (processes) cannot be identified independent of both contents and products. That is, even if one attempted to assess the simplest information processing elements (e.g., simple and choice reaction time (RT)), the stimuli must exist within some content—whether it be figural/spatial (lights or auditory signals), symbolic (e.g., numbers or letters), semantic (words or sentences), and so on.

Similarly, one cannot assess the content components of intelligence (e.g., verbal, spatial, numerical) without some kind of operation or process. Even the most basic vocabulary test item (Define ____) requires oral comprehension (if the test item is presented in an auditory format) or reading comprehension (if the test item is printed), and further requires memory retrieval processes and response processes in order to answer the question. As such, every test item will represent a combination of content and processes, rendering the question ‘Is this a process test item or a content test item?’ impossible to answer in a definitive fashion.

However, even though such a separation of content and process is problematic at a fundamental level, in practical terms, test items can clearly be identified as more highly associated with process or more highly associated with content. The identification is largely accomplished by the choice of stimuli for the test content and the nature of the processes required to answer the test questions. Most notably, the experimental psychologists’ approach to assessing ‘elementary cognitive tasks’ (ECTs, see Carroll 1980) is to construct items of highly familiar stimuli (e.g., numbers, letters, and simple figures or polygons), or highly novel stimuli (i.e., stimuli that would be expected to be unfamiliar to the majority of test takers). Performance on a choice RT task that uses only numbers, letters, dots, or common symbols (such as punctuation marks) is expected to be generally unaffected by differences among these different contents. Evidence from basic research into this question largely confirms this assertion, in that there are high correlations between choice RT tasks with numbers, letters or symbols as stimuli—see, for example, Ackerman (1986) and Kyllonen (1985). In contrast to process-dominated measures, content-dominated measures are those that are largely identified as tapping some particular knowledge or developed skill, such as the definition of a word, the identity of a familiar shape (e.g., a square or a circle) or object (e.g., a photo of the Eiffel Tower, the Leaning Tower of Pisa), a fact about the world (e.g., the distance between New York and London; the number of states in the USA; why helium balloons float), and so on.

## 3. A Brief History of Modern Intelligence Tests with Respect to Process and Content

In order to understand why both process and content measures tend to be included in omnibus intelligence tests, a review of some historical developments in assessing intelligence is needed. A selective review follows of the salient developments in intelligence assessment, with respect to these kinds of measures.

### 3.1. Galton (1883) and Spearman (1904)

From a historical perspective, the contrast between assessing intelligence with a concentration on process or a concentration on content is more straightforward than defining the two constructs. Galton (1883), for example, focused on the assessment of individual differences in sensory and perceptual processes, but did not compare these measures with one another or with other assessments of intelligence. The prevailing conjecture, though, was that because information was received via sensory and perceptual processes, then those individuals with superior sensory and perceptual process abilities would be certain to be those with high intelligence. However, two early studies (Cattell and Farrand 1896; Wissler 1901) reported essentially null results when examining associations between process measures and other assessments that purported to represent intelligence (such as academic grades).

Spearman (1904), in his seminal article on general intelligence, reported on a series of studies that involved the assessment of “discrimination” and intelligence, and of their intercorrelations. The tests of discrimination were classic assessments of psychophysical processes, such as auditory pitch discrimination, visual discrimination of the shades of cards, and tactile discrimination of a series of weights. The assessments of intelligence consisted of the rank order of school grades, ratings of intelligence by teachers, and peer ratings of intelligence. Spearman reported (p. 269) that the correlations between assessments of discrimination and assessments of intelligence, after correction for unreliability, were essentially *r* = 1.0—that is, a complete overlap between them. It would be difficult to assert that his assessments of intelligence were measures of ‘content’, but Spearman’s assertion was that the measures of basic psychophysical processes provided the same rank-ordering of individuals as the various ratings by teachers and peer students on intelligence, *if* one corrected for the unreliability of the measures. Spearman concluded that:

“... there is also shown to exist a correspondence between what may provisionally be called “General Discrimination” and “General Intelligence” which works out with great approximation to *one or absoluteness*.”

(p. 284)

### 3.2. Ebbinghaus (1896–1897)

Although the purpose of the study by Ebbinghaus was to assess mental fatigue among schoolchildren, as an effort toward determining the recommended maximum length of instruction during the school day, the assessment he developed (The Completion Test) turned out to be highly correlated with individual differences in school grades. The test was constructed of a text passage. The passage was provided to the students on paper with omissions of words and word fragments, and blanks in their places. The task of the examinee was to fill in the blanks with the missing material, based on predictions made from the context of the text itself, which makes this a verbal-fluency content measure.

In a later version of the test, Terman (1906) first had the examiner read the text aloud, and then the completion test was administered. Variants of this test can be found in many modern intelligence measures or as stand-alone ‘cloze’ or ‘completion’ tests (for a review, see Ackerman et al. 2000). If the test is administered in the fashion described by Terman (1906), it represents a combination of ‘memory’ abilities and ‘fluency’ abilities, with an unknown level of contribution from each of these process and content abilities. However, if the test is administered without first reading the text aloud, then the test is most certainly highly associated with fluency (content) abilities.

### 3.3. Binet and Simon ([1905] 1973)

Most intelligence researchers are well aware of the justification for creating the first modern intelligence scales, so this part will be brief. As described by Binet and Simon (e.g., see Binet and Simon [1905] 1973, trans by E. Kite):

“In October, 1904, the Minister of Public Instruction named a commission which was charged with the study of measures to be taken for insuring the benefits of instruction to defective children. They decided that no child suspected of retardation should be eliminated from the ordinary school and admitted into a special class, without first being subjected to a pedagogical and medical examination from which it could be certified that because of the state of his intelligence, he was unable to profit, in an average measure, from the instruction given in the ordinary schools.”

(p. 9)

The “psychological” scales developed by Binet and Simon ([1905] 1973) were designed to augment these other assessments for establishing whether children should be removed from the mainstream classroom and placed in some form of training or special education more suitable to their mental capabilities. A review of these scales finds that they have a mixture of process and content components. Process components, for example, include ‘judgment’ (‘weight comparisons’), ‘memory’ (‘repetition of objects‘), and ‘suggestibility’, while content components, for example, include ‘verbal knowledge of objects’, ‘recognition of food’, and ‘naming of designated objects’. Unfortunately, for the current intents and purposes, Binet and Simon did not provide any separate information regarding the relative validity of the individual scales—their assessment yielded only an aggregate ‘mental age’ score, which could be compared to the child’s chronological age. When a child had a mental age substantially (e.g., 2 or 3 years) lower than the child’s chronological age, the child was diagnosed to be ‘retarded’.

As noted by Binet and Simon, the intelligence scales were not to be regarded as *definitive* indicators of whether the child should be removed from the mainstream classroom and placed in a special education situation; rather, their psychological scales were to be considered in the context of medical and pedagogical assessments of intelligence (where the pedagogical assessments were mainly ‘content’ assessments of knowledge and skills acquired through school instruction and from outside experiences), along with other qualities of “attention, will, regularity, continuity, docility, and courage which play so important a part in school work, and also in after-life; for life is not so much a conflict of intelligences as a combat of characters”. (Binet and Simon [1905] 1973, p. 256).

In Binet and Simon’s writings, it is clear that intelligence assessments served (as part of an overall evaluation) both ‘rule-in’ and ‘rule-out’ purposes. The rule-in purpose was to detect mental retardation in conjunction with the other assessments. The rule-out purpose was to identify children who were of normal or near-normal levels of intelligence, who may have been mis-diagnosed by the pedagogical method, the medical method, or teacher referrals (e.g., when a teacher might mis-identify an inattentive or troublesome student as retarded). Thus, the criterion for the Binet–Simon scales was the determination of whether the child would benefit from mainstream classroom instruction, or would be better served by a special educational placement. However, it is especially important to note that Binet and Simon regarded intelligence as a concept to be potentially variable, and not fixed. They noted that follow-up tests might reveal changes in the individual’s relative scores (with respect to their similarly chronologically-aged peers), that would imply a different placement; that is, either moving the child from special education to the mainstream classroom or moving the child from the mainstream classroom to a special education placement.

With this original conceptualization of intelligence assessment as the initial background, one could propose that criterion-related validity should be evaluated in terms of the valid determination of an ‘ability to benefit’ from mainstream classroom placement, and make the determination of the comparative validity of the process and content components, in terms of a signal-detection paradigm (considering relative true and false positive and negative diagnosis proportions). Yet, such statistics are entirely elusive from the historical record.

## 4. Translations and Revisions of the Binet–Simon Scales

The purpose and scope of assessments of intelligence, however, did not remain static in the years following the introduction of the Binet–Simon scales. In the United States especially, several individuals created English translations (and expansions) of the Binet–Simon scales, and most fundamentally, changed both the underlying theory and purposes that underpinned the Binet–Simon scales. The two most notable influences in this domain were H. H. Goddard (1914) and L. M. Terman (1916). Goddard suggested that the Binet–Simon scales should be used to institutionalize ‘mental defectives’, instead of having them in mainstream educational placements. Though Goddard did recognize that other indicators were relevant, he suggested that the medical assessment of intelligence was not useful, and he had nothing to say about the pedagogical method of assessing intelligence. Binet and Simon recommended specific curricular plans for children placed in special education (e.g., “they should be given lessons of well, of attention, of discipline; before exercises in grammar, they need to be exercised in mental orthopedy; in a word they must learn how to learn”, Binet and Simon [1905] 1973, p. 257.). Goddard, in contrast, focused on either obtaining work permits for the children or perhaps training them to be productive by working on tasks that could generate income while relegated to institutions for the ‘feeble-minded’. Moreover, Goddard’s views on the source of individual differences in intelligence were strongly hereditarian, with the implication being that one’s placement on a continuum was largely fixed and not subject to change. Goddard did not have much to say about process vs. content components of the intelligence assessment, and appeared to have been entirely satisfied with a unitary score to represent relative standing on the intelligence assessment.

Terman’s influences on the nature of intelligence assessment and the uses of such measures were much more profound than Goddard’s, even though they shared opinions on the heredity and fixed nature of one’s relative intelligence. In particular, Terman was interested in not only identifying cases of mental retardation, but also identifying individuals at the higher end of the intelligence continuum, with the goal of differential placement of “genius” students outside of the mainstream classroom. From an assessment perspective, Terman translated and expanded the Binet–Simon scales, most notably by attempting to make more precise assessments of ‘advanced’ levels of intelligence. Terman also diverged from Binet by adopting Stern’s (1914) notion of an intelligence quotient (mental age divided by chronological age; though Terman multiplied the quotient by 100 to yield a ‘normal’ IQ score of 100), which by implication identifies relative standing on an intelligence test to be a fixed, unchanging value. Terman reported that the correlation of the overall Binet–Simon test and teacher ranking was *r* = .48 (Terman 1916, p. 75).

## 5. Criterion-Related Validity

### 5.1. Predicting Academic Success of Children

One of the most vexing difficulties in evaluating the criterion-related validity of intelligence assessments is the lack of recent data on the underlying criteria. The original purpose of intelligence testing was at least partly a consequence of the introduction of universal public education, where a method was needed to predict which students would not successfully complete academic coursework in the mainstream classroom. The Binet–Simon scales, along with various translations and adaptations, were developed to objectively diagnose intellectual deficiency, or ‘feeble-mindedness’ (in that time). The implication of such a diagnosis was that such students should be removed from the classroom. In the late 1800s and early 1900s, removal from the classroom for intellectual deficiency typically meant that the child was either put into a special class for ‘feeble-minded’ students (the precursor to some types of ‘special education’) or committed to an institution that focused on ‘feeble-minded’ children (e.g., the “Vineland Training School for Feeble-Minded Girls and Boys”, the “School for the Feeble-Minded, Faribault, Minnesota”, etc.). Thus, the initial criterion-related validity of an intelligence assessment would be reflected in whether a child fails or succeeds in the mainstream school classroom.

Subsequent developments in intelligence assessment for children—especially identified with Terman and the Stanford–Binet scales (e.g., Terman 1916), substantially expanded the scope of the criterion to accommodate this interest in identifying both low and high intelligence individuals. From this perspective, the academic performance criterion for intelligence assessments evolved to include the prediction of academic grades, that was beyond a determination of failure/success in the mainstream classroom.

But the general tone of Terman’s position was that the IQ test should be the *criterion*, rather than a predictor of school performance, in some sense, turning the Binet–Simon perspective on its head. That is, rather than *predicting* school failure from the IQ test, it was to be used for validating the reasons for school failure. Terman (1919) asserted that students who performed poorly in school or performed exceptionally well in school should be administered an IQ test, and based on the test results, the student should be removed from the classroom or placed in a lower grade (if the IQ was low), or advanced in grade to match the child’s mental age to the chronological age of the class members (if the IQ was high).

At this point, one is tempted to throw up one’s hands in bewilderment. How can one hope to validate a measure of intelligence, when the measure itself is the criterion, not the predictor of some other variable? The explicit statements from such a perspective are that there are two potential sources of error in the alignment between the intelligence test score and academic success: (1) subjectivity or error on the part of the teachers in assigning grades, and (2) a list of other sources that might affect school success (e.g., interest, personality, moral tendencies, etc.). Neither of these types of influences has any value in determining the validity of the intelligence test for academic success.

### 5.2. Selection for College/University Study

Ultimately, the main reason why intelligence tests in the USA in particular were validated against academic success appears to have resulted from the development of measures for predicting post-secondary school grades. Several tests were developed during the 1910–1920 period, including Thorndike’s College Entrance Tests (Thorndike 1920, 1921), Thurstone’s Intelligence Test IV (Thurstone 1919), the Terman Group Test of Mental Ability, versions of the Army Alpha test (see Yerkes 1921), and the Stanford–Binet. As reported by Toops (1926), by 1924, 60% of surveyed universities had adopted some form of intelligence testing, while an additional 12% were trying out using the tests experimentally. Terman (1921) reported a sample of validity correlations for the intelligence tests against grades in university study, with typical results in the *r* = .4 to .5 range. However, none of these reported results made any finer distinction than overall IQ or general intelligence scores as predictors of academic success.

The positive aspect of these results is that, at least for post-secondary applications, the intelligence tests were mainly considered as predictors of academic success, rather than criteria in and of themselves. The main exception to this point is that several early investigators proposed administering intelligence tests to students who faced academic failure, in an effort to determine whether they should have not been admitted to college/university study to begin with.

## 6. Wechsler Scales

In terms of providing a comparison and contrast between process components and content components of an intelligence assessment and their respective predictive validities for academic performance, the earliest systematic approximations come from investigations with the Wechsler scales. The Wechsler–Bellevue scales were first introduced in the late 1930s as an intelligence assessment for adults, and were modeled on the Binet–Simon, the army tests from World War I, and other similar sources (Boake 2002; Wechsler 1939). Although these scales were not originally designed for predicting academic success, the structure of the test makes is possible to at least provide a rough contrast between tests that are primarily associated with content (the Verbal subscales) and process (the Performance subscales). From a task-analytic comparison, it is reasonable to assert that the Verbal subscales while dominated by content-oriented scales (e.g., Information, Similarities), also had some tests that have process components assessed (e.g., Digit Span, Arithmetic); but that the Performance subscales had limited content components and were dominated by process-oriented components (e.g., block design, object assembly).

In a small initial study of 30 University of Arizona students, Stevenson (1952) reported that the Verbal composite scale correlated with university grades *r* = .36, while the Performance composite scale correlated *r* = .12 with grades. The full-scale IQ (which is a composite of both Verbal and Performance subscales) correlated *r* = .35 with university grades—virtually the same correlation as obtained with just the Verbal composite. It may be of further interest to note that the Wechsler scales performed more poorly in predicting grades when compared to the Ohio State University (OSU) Psychological Test (*r* = .69 with grades). The OSU Test was composed of three subtests (Same and Opposites, Analogies, and Reading Comprehension), which would fall mostly onto the ‘content’ side of the process-content continuum. However, given the small sample, it is important to interpret these results as no more than suggestive that there is an advantage of content measures over process measures for predicting college grades.

A subsequent study with larger samples and the revised version of the Wechsler scales (the Wechsler Adult Intelligence Scale, WAIS) was reported by Conry and Plant (1965). The study included the prediction of grades with a sample of N = 98 high school students, and a prediction of grades with a sample of N = 335 college students. The validities of the WAIS Verbal and Performance composites were reported as *r* = .63 and .44 for the high-school sample, respectively, and *r* = .45 and .24 for the college students, respectively. The authors reported correlations for the individual WAIS scales, in addition to the composite scores. Interestingly, all of the verbal scales (except Digit Span, which is arguably more of a ‘process’ measure, anyway) correlated substantially (*r* = .45 to .65) with grades in the high-school sample, but none of the Performance scale validities were above *r* = .34. The same general pattern was found for individual scale prediction of college scores, with a somewhat reduced overall level of validity; a result that is entirely consistent with expectations of a restricted range-of-talent in the college sample, compared to the high school sample.

## 7. Modern Intelligence Tests

With the exception of the Cognitive Assessment System (CAS 2, Naglieri et al. 2014), most omnibus modern intelligence tests (e.g., Stanford-Binet, Roid 2003; e.g., Wechsler, Wechsler 2014; KABC-II, Kaufman and Kaufman 2018) have adopted some version of the Cattell–Horn–Carroll (CHC) model of intelligence in selecting various test subscales, in order to capture process components (e.g., fluid intelligence; *Gf*) and content components (e.g., crystallized intelligence; *Gc*) in the overall computation of a composite intelligence (or IQ score) or in the reporting of separate composites, such as the traditional verbal and performance composites associated with various versions of the Wechsler scales. The CAS 2 stands out in that it purposely ignores the content components of intelligence and focuses exclusively on process measures.

Yet, none of these modern test developers report criterion-related validity for school grades, either for omnibus IQ measures or for individual measures of process/content or their respective composites.

## 8. Some Specific Characteristics of Process and Content Measures

### 8.1. Process Assessments

A large number of processes have been proposed as possible essential indicators of individual differences in intelligence. In general, it is probably a reasonable assertion that there is a high correlation between the amount of time it takes to complete one trial of a process assessment and the level of complexity of the process being assessed (e.g., see Kyllonen 1985). At the lowest level of complexity, these include simple and choice reaction time (RT) measures (or the slope of RT as a function of the number of choices in choice-RT measures, e.g., see Jensen and Munro 1979), and ‘inspection time‘ measures, which involve comparisons, for example, between line lengths between two items shown with brief exposure times (e.g., see Kranzler and Jensen 1989; Vickers et al. 1972).

At more intermediate levels of complexity, process measures include the Posner Task (involving comparisons of physical or lexical identity of letter stimuli) and the Sternberg memory scanning task (where a memory set of objects (typically letters or numbers) is presented, followed by brief display of letters, for which the examinee must determine whether the displayed items include a stimulus from the memory set). Short-term memory processes, such as the kind of assessment that is common on traditional IQ tests (e.g., forward or backward digit span) are more complex than simple or choice RT tests.

Increasing the level of complexity process measures with stimuli beyond individual letters and numbers typically involves even more knowledge of the categories of items (e.g., recognizing that a ‘dog’ is a member of a category of four-footed animals), or the meaning of relationships between objects, as in the sentence verification task, where a star is placed above or below a cross in the first stimulus and the examinee is required to determine the accuracy of a written sentence that follows (e.g., STAR BELOW CROSS).

Higher levels of complexity of process assessments are associated with measures of executive function (e.g., Arffa 2007), working memory (e.g., Wilhelm et al. 2013), and some spatial abilities (such as Speeded Rotation and Spatial Orientation, see Lohman 1979, 1987). While each of these ordinarily involves highly familiar or highly novel stimuli, the solution times are typically much longer than for the assessments of low or intermediate levels of process complexity. In addition, the cognitive load for solving such problems increases exponentially, such that in many examples, completion time is no longer used as the measure of performance, but rather accuracy (correct or incorrect answers) is assessed as the indicator of process ability.

At the highest level of complexity, process assessments often resemble subtests on existing IQ-type measures. Spearman, although he initially regarded the Ebbinghaus completion test (a mostly content-dominated assessment) as the indicator with the highest loading of any measure on *g* (see Krueger and Spearman 1907), ultimately settled on the inductive reasoning measure developed by Penrose and Raven (1936) and later known as Raven’s Progressive Matrices (Raven et al. 1977), as the essence of *g* (Spearman 1938). Other measures of deductive and inductive reasoning, such as Cattell’s so-called Culture Free (or Culture Fair) Intelligence Test (Cattell 1940) and measures of spatial visualization (e.g., the Shepard–Vandenberg rotation test; see Eliot and Smith 1983) are other exemplars of high-complexity process-dominated assessments.

### 8.2. Inherent Problems with Process Ability Measures

There are two related problems associated with process-ability measures inherent to the underlying theory and composition of such tests. The first problem is one of familiarity, for tests designed to assess a construct that involves novel processes, and/or processes with novel item content. This is accomplished, either by introducing operations for which the test-takers are unfamiliar, and/or by requiring the processing of unfamiliar stimuli. Such process-based tests may in fact be appropriate, but if there is variability in familiarity with the processes or stimuli, performance levels on the test may be confounded by individual differences in the degree of familiarity. The familiarity may range from a broad transfer-of-training (e.g., from experiences of the test-taker playing similar cognitively demanding games outside of the testing situation) to narrow transfer-of-training, or ‘test coaching’, where the test-taker is given exposure to the actual stimuli and/or processes required by the test itself. In either case, such opportunities can be expected to provide performance advantages to those with test-related experiences. Transfer-of-training experiences appear to yield aptitude–treatment interactions, in the sense that higher-ability individuals may benefit disproportionately from far-transfer experiences, while lower-ability individuals may benefit disproportionately (e.g., see Sullivan and Skanes 1971).

The second problem relates to the ‘learnability’ of the processes demanded by the test. At a surface level, learnability may involve developing familiarity with the stimuli involved in the test items. As such, exposure to item content, along the lines similar to test coaching may be sufficient to reorder individual differences in test performance. However, for the underlying processes, it may be that skill may be developed for those operations. Most investigations along these lines focus mainly on repeated testing under the same conditions for each test. There is more than adequate empirical evidence (e.g., see Rockstroh and Schweizer 2009), that ‘mere practice’ improves performance on such tests, even in the absence of direct instruction or coaching. However, most learning studies are designed so that the performance improvements are maximized by providing direct *instruction* along with practice. Feedback and knowledge-of-results have been found to yield much larger gains in performance over repeated trials in most tasks, compared to mere practice. Moreover, regardless of whether the processes required by the tasks are simple or complex, there are declining correlations between the initial performance and post-practice performance, as the number of trials of practice increases (e.g., see Ackerman 1987; Humphreys 1960).

The implication of these considerations is that there are fundamental differences between process-oriented tests administered to test-takers who are completely naive to the stimuli and/or process requirements of the tests and those who have had either direct learning/skill-acquisition experience or indirect (through transfer) experience with the stimuli or processes. Contrary to Thorndike’s (1908) assertion that practice and learning effects on such tests will result in increasing between-individual variability in performance (and, thus higher associations with other measures of intelligence), the vast majority of studies indicate that the exact opposite occurs—that is, between-individual variability typically shows a substantial reduction with increased learning/practice opportunities, and the correlations between post-practice performance on such process tasks and other measures of process-oriented tests and measures of general intelligence are lower than pre-practice performance assessments. In this sense, there is an intrinsic contradiction between the assertion that ‘pure’ measures of processes are highly associated with intelligence, because the provision of learning/practice opportunities can be assumed to reduce the effects of prior experiences and exposure to the particular test demands, and only then can the test be validly claimed to represent asymptotic process performance differences between individuals. Perhaps the only sensible argument against the position taken above is that the ‘process’ the test developer intends to assess has as its core property, the *processing of novel stimuli*. The problem with this position, however, is that there is no insurance that individual test-takers do not differ in the experiences that lead to transfer-of-training/knowledge from other tests/tasks to the process test under consideration.

### 8.3. Content Assessments

The most straightforward examples of content measures are those that are composed of knowledge assessments. As such, they would be well-placed in the *Gc* domain. The simplest content measures are those of recognition—for example, a vocabulary test item that presents an examinee with a target word and a definition, for which the examinee must decide whether the definition suits the target word. Similar tests could be constructed for numerical content (is ’13‘ and odd or even number?) or spatial content (is the pictured shape a square?). However, content measures on early IQ-type tests required not recognition, but recall. Instead of asking an examinee to recognize the correct answer, the examinee might be asked to define a particular set of words. Ceteris paribus (all else being equal), recall is traditionally considered to be more difficult than recognition. In fact, one of the criticisms of the shift from one-on-one intelligence assessment to group testing formats was the reduction of cognitive demands involved in shifting from open-ended recall items to multiple-choice recognition items (e.g., see Carroll 1982). Nonetheless, recall items persist in modern one-on-one IQ-type tests.

More generally, knowledge and skill assessments may involve much more complex and extensive learning and practice. Tests of numerical abilities that require addition, subtraction, multiplication, and division, or even more complex problems of algebra and geometry are first and foremost content tests, but they have a non-trivial involvement of process demands as well. An addition test item that requires an examinee to add several numbers together without writing down the intermediate results will involve the content of the skills for performing addition, along with the processes of keeping track of the sums when adding down a column of numbers. Consistent with the Cattell framework, the degree to which test performance is more highly associated with *Gc* or *Gf*—that is, content or process, may depend on the prior learning history of the examinees. For example, if an item asks for the square of 12 or the square root of 144, an adult might simply recall the answer from prior knowledge; but a child or adolescent might need to work out the answers by hand (e.g., multiplying 12 × 12 or trying out increasing numbers to determine the square root, respectively). These considerations make it especially difficult to determine the relative influence of content and process on intelligence test assessments.

The traditional approach to classifying tests as achievement or intelligence tests usually rests on the explicit exposure to instruction and learning. Questions of geography, for example, that are administered to students at the end of a course on the topic would be considered achievement tests if the students were explicitly exposed to the information as part of the curriculum. The question, ‘What is the distance from New York to London?’ in that context might reflect a fact directly communicated to the students that only needs be recalled accurately for the examinee to provide a correct response. In the absence of explicit instruction, the determination of content vs. process may be idiosyncratic for individual examinees. One examinee might have learned this fact from sources at school or at home, while another might not know the answer outright, but be able to make an estimate from various other known sources of information (e.g., a recollection of how long such a trip takes, and then a calculation based on knowledge of airspeed of a commercial airliner; or knowledge of the time difference between London and New York, and an estimate of the span of time zones at their respective latitudes). The estimations clearly involve both content knowledge and a variety of problem-solving processes.

## 9. Speed vs. Difficulty

There is no *fundamental* reason that content-oriented intelligence tests focus mainly on item difficulty with a reduced emphasis on the speed of item solution, and most process-oriented intelligence tests vary considerably in terms of the emphasis on speed and difficulty. Although the first modern intelligence tests (Binet–Simon and various translations) had reduced emphasis on speed of responding, which is followed, more or less, by current one-on-one intelligence tests, some researchers theorized that the underlying construct of intelligence had the speed of solving problems as a key contributing factor. For example, Thorndike (1924) proposed that there are three elements to intelligence: difficulty, extent, and speed. Specifically, he argued that even if two individuals can correctly answer the same test items, the individual that provides the answers more quickly has greater intelligence. For content-oriented intelligence tests, the speed of processing became a more integral aspect of intelligence assessment mainly when group testing was introduced (e.g., in the mid-1910s with the Army Alpha and Beta tests), even for content-oriented intelligence tests, arguably more for efficiency of testing reasons than for underlying theoretical justifications.

Extensive research on this topic was reported up to the mid-20th century, with results indicating that even when content-oriented tests were varied in their speed requirements, identifiable ‘speed’ ability factors could be determined along with content factors, such that they appear to represent different underlying intellectual abilities (difficulty and speed, respectively). There remains some uncertainty as to whether the speed factors found in one domain (e.g., verbal ability) are the same or related to speed factors found in other domains (e.g., spatial, math abilities). Davidson and Carroll (1945) identified a general speed factor across tests, but Horn (1965) identified a higher-order factor of General Speediness.

Many early and current process-oriented intelligence tests, however, have speeded processing as an integral, if not the central determinant of performance. Tests like the digit-symbol test, like many other perceptual speed assessments, are trivial to perform with a high degree of accuracy under unspeeded conditions. That is, most test-takers are capable of a nearly perfect performance if the testing time limits are long; the key assessment is how many items are correctly answered with time-limits so short as to prevent nearly all test-takers from completing all of the items. Similarly, process-oriented tests may be only, or nearly completely tests of speed, such as simple and choice RT tests; which is characteristic of both psychomotor ability tests and those that purport to measure the speed of information processing.

## 10. “Indifference of the Indicator”

Although Spearman (1904), was arguably one of the most substantial contributors to the theory of intelligence, he made two fundamentally wrong-headed assertions: (1) that there were no identifiable ‘group factors’; that is, the only common variance between any two measures of intelligence could be attributed to their shared variance with *g*—an assertion that has been roundly and effectively criticized for the past 100 years (e.g., Kelley 1928; Thurstone 1935; etc.), and (2) that “for the purpose of indicating the amount of *g* possessed by a person, any test will do just as well as any other, provided only that its correlation with *g* is equally high” (Spearman 1927, p. 197); that is the principle known as the “indifference of the indicator”. Spearman’s assertion may hold for estimates of his *g* construct (which is largely a theoretical question, given the abstract nature of the *g* construct in his framework), but there are no external criteria specified against which to validate this representation of intelligence.

In essence, it is ultimately silly to generalize the “indifference of the indicator” conjecture to real-world assessments of intelligence. From a conceptual perspective, the point is well illustrated by the Wittmann and Süß (1999) Brunswik Symmetry perspective, in that when the criteria are not extremely broad, the most suitable (and most highly valid) predictors of performance will match both the breadth and content of the performance criteria. From a practical perspective, it is intuitively obvious (and empirically demonstrated; e.g., see Ackerman et al. 2013) that, for example, a robust test of math abilities will be a more effective predictor of individual differences in science, technology, engineering, and math academic achievement than, say, a robust test of verbal abilities, for which the math and verbal tests have equivalent correlations with a general ability composite or IQ measure.

## 11. Standardized Achievement Tests as Criteria for Intelligence Assessments

It can be argued that standardized achievement tests—typically those designed to be administered to students across schools, counties, and states—have advantages for validating intelligence tests over pass/fail determinations in an individual school, or grade point average (GPA). There are two main reasons for this argument, that is: (1) the tests are objective, and therefore less likely to be influenced by subjective judgements by teachers, and (2) they are typically tied directly to the curriculum of a specific grade, in terms of content validation. However, there are several limitations of standardized achievement test measures, as follows: (1) They represent a very small sampling of the individual student’s behavior—a few hours in comparison to the cumulative accomplishments of the student over the course of a year; (2) Because they are often high-stakes situations, achievement tests tend to emphasize maximal performance, rather than typical performance. This means that there is a common method to both the intelligence tests and the achievement tests, that may artificially increase the correlations between the two, as a function of common (situation) method variance; (3) The format of achievement tests typically involves multiple-choice questions, which usually require recognition, rather than recall of content; (4) Because the achievement tests are typically administered as group tests, they require reading skills, including both the test instructions and for some domains, the content of the test. As noted by Carroll (1982), there is an implicit assumption that individual differences in reading skills are orthogonal to the content or process abilities that the test aims to assess; an assumption that is likely not true. Thus, standardized achievement test scores are likely to be confounded by individual differences in reading skills.

Are there differential implications for process-oriented or content-oriented intelligence assessments when validated against standardized achievement tests? To the degree that standardized achievement tests are themselves assessments of content, namely declarative knowledge, the premise is that intelligence tests with overlapping content would likely have higher validity than process tests. In contrast, in standardized achievement tests in the domain of math, because they focus more on the procedural skills than on content knowledge, higher validity might be relatively more highly associated with overlapping process-oriented intelligence assessments. The main qualification to this expectation is that few process-oriented intelligence tests actually have overlapping content because they focus on novel processes rather than well-learned processes, such as computational arithmetic or more advanced processes, such as algebra, geometry, or calculus. A review of this particular literature is beyond the scope of the current paper. However, consistent with the principle of Brunswik Symmetry, the general sense is that when there is an overlap of content (e.g., math abilities and math achievement, reading comprehension ability, and content-based literature achievement), there is a much higher corresponding correlation than when there is less content overlap, and that process measures overall, have much lower validity for standardized achievement tests than content-overlapping intelligence assessments.

## 12. Meta-Analytic Results

The ideal study for evaluating the relative contributions of content and process intelligence measures for the prediction of academic success would involve measures of each type to be administered to the same sample at the same time. Moreover, there would be multiple measures of each construct (e.g., see discussion by Ackerman and Hambrick 2020), in order to average across any test/item-specific idiosyncrasies of the individual tests (e.g., method variance; see Humphreys 1962). From such a design, and information about the respective reliabilities of the individual scales, it would be possible to generate estimated true-score validity correlations for each of the content and process measures. The two studies that involved administration of the full Wechsler measures discussed above partially meet these specifications, in that they involve a single sample of participants completing both content and process intelligence subtests, but there remains an under-determination of the respective underlying contents and processes that have been hypothesized to be the critical contents and processes of intelligence.

At the level of individual studies, it is impossible to determine whether content or process measures are more highly valid predictors of academic success, if the study fails to include measures of both content and process components of intelligence. For example, assume that one study examined a content ability test (e.g., vocabulary or knowledge about science) and obtained a particular correlation with academic success, and another study examined a process ability test (e.g., working memory or choice reaction time), with a different sample of students, and also obtained a correlation with academic success. How should the results be interpreted? If one correlation is significantly larger than the other, what could be concluded? The answer appears to be that virtually nothing could be concluded, first, because the tests would likely differ in terms of reliability. Corrections for reliability could be made, but then what of the sample differences? If the samples were matched—how should they be matched—perhaps full-scale IQ? However, if full-scale IQ measures were available, then the problem of incommensurability would be avoided from the very beginning. Other differences would presumably include conditions of testing, prior selection, different populations (e.g., different kinds of schools, grade levels, other relevant demographic characteristics, and so on). Finally, the choice of *which* content and *which* process measures to include is also critical. A difference in the suitability of measures (e.g., using Verbal content measures to predict math or science grades) would similarly render comparative differences between content and process validities, largely meaningless. Thus, any individual study that lacks simultaneous administration of samples of both content and process measures are unlikely to be conclusive about the respective validities.

It is possible, though by no means certain, that meta-analytic techniques could be used to avoid some of these threats to valid comparisons. That is, if individual studies of process or content intelligence measures were randomly distributed in the literature with respect to key variables of the sample characteristics, the suitability of the intelligence test measures, and so on, with a suitable number of studies, and appropriate statistical adjustments for test and criterion reliability and sample differences, some conclusions might be reached about the relative merits of content and process measure validity for predicting school grades.

The closest approximation to the ideal situation in the literature to date is a meta-analysis by Roth et al. (2015). While still quite limited (in that these authors only categorize intelligence tests by “Verbal” and “Nonverbal”), they were able to identify 59 coefficients from studies with verbal tests and 89 studies with nonverbal tests, with samples limited to primary and secondary school students. The verbal intelligence tests included the Wonderlic Personnel Test and several broad verbal reasoning and vocabulary tests. The nonverbal tests were dominated by Raven’s Progressive Matrices, Cattell’s Culture-Fair Intelligence Test, and similar abstract reasoning measures. For scales that are most often used in educational settings, such as the Stanford–Binet and Wechsler, there were surprisingly few studies found. Moreover, of the 17 studies with such measures, nearly half of them were published 50–92 years ago, well beyond what might be considered the useful lifespan of a criterion-related study, given the changes in the schools, their populations of students, and in the intelligence assessment instruments themselves.

Although the Roth et al. (2015) meta-analysis did not include the high- school student data from the Conry and Plant (1965) study described above, the pattern of meta-analytic results reported by Roth et al. (2015) were largely consistent with those of Conry and Plant. The estimated true-score validity for the verbal tests was (rho) = .53, and the estimated true-score validity for the nonverbal tests was (rho) = .44. The validity for nonverbal tests was, in essence, generally significantly lower for the nonverbal tests than the verbal tests. Given the qualifications about the suitability of the various tests, and noting that the range of contents and processes assessments that make up the corpus of tests, the general sense of these various sources is fundamentally that there has *not* been a demonstration that process intelligence measures are as valid as content tests are for predicting academic success, whether in primary or secondary school (and some indication that this also holds for post-secondary school success).

## 13. Clinical Diagnosis

Ever since the introduction of the Wechsler–Bellevue scales (Wechsler 1939) with the presentation of separate Verbal and Performance composite scores, along with an overall IQ score, there has been a lively discussion, or perhaps it is better described as a ‘contentious’ controversy regarding the validity of such scores for making a variety of differential clinical diagnoses (e.g., see Kaufman 1994). Because the current paper is focused on the validity of IQ-type measures for predicting academic performance, this discussion is largely beyond the scope of the inquiry. Perhaps the most robust justification for considering process vs. content distinctions in the clinical diagnosis domain is the theory first put forward by Hebb (1942), which was appropriated by Cattell (1943)—see Brown (2016) for an account of the history. Basically, Hebb described two domains of intelligence, where one source of intelligence was fundamentally associated with ‘processes’, designated as Intelligence A (e.g., reasoning, learning), and the other was associated with ‘content’, designated as Intelligence B. Hebb’s framework, based on a neurological perspective, was based on the examination of clinical patients who experienced neurological incidents (stroke, epileptic seizures) early vs. late in life. Those patients who experienced these incidents late in life tended to have impairments in Intelligence A—processes, and were more likely to retain their Intelligence B—content knowledge and skills. Those who experienced neurological incidents during childhood or adolescence were more likely to have broad impairments—both Intelligence A and Intelligence B, suggesting that Intelligence B forms out of Intelligence A; a concept later adopted by Cattell in describing *Gf* and *Gc*, and proposing that *Gc* grows via an ‘investment’ of *Gf* processes in acquiring content knowledge and skills (Cattell 1957).

Although Hebb was mainly concerned with adult intellect, one possible implication of his framework is that individuals who had relatively lower levels of Intelligence A (or *Gf*) would be expected to have poorer prospects for future academic achievement (i.e., development of Intelligence B/*Gc*), compared to individuals with higher levels of Intelligence A/*Gf*. This would suggest that process assessments should be more highly valid predictors of academic success, compared to content assessments. However, the critical counter-argument to this inference is perhaps best articulated by Ferguson (1954, 1956). He suggested that, except for the newborn infant, the most important determinant of individual differences in new learning is the transfer from existing knowledge and skills. In that context, one would thus expect that content abilities would be more influential predictors of academic success than process measures, especially when considering students in advanced grades, namely adolescents and adults—as their respective repertoire of knowledge and skills will be more robust than students early in the first few years of school. From a pragmatic perspective, modern selection tests for college/university admissions have entirely separated from the intelligence-labeled assessments of the 1920s (e.g., the Army Alpha), and have evolved to become mainly content-dominated assessments, especially as they have shed the more process-oriented subtests such as analogies. Yet, it is important to note that although there have been no successful demonstrations that process-dominated intelligence measures have utility for selection in predicting post-secondary school success, either in isolation or in comparison to content-oriented tests, it remains *possible* that such an investigation might at least provide useful data to make a comparative determination of the relative merits of process and content intelligence measures.

## 14. Child/Adolescent vs. Adult Intelligence

A reviewer and the editors of this Special Issue requested that the treatment of process and content aspects of intelligence be expanded to include considerations of aging and adult intelligence. Doing so requires that one expand the consideration of the external validity of intelligence in terms of both academic performance and non-academic domains, because the prediction of post-secondary academic performance presents challenges well beyond that of predicting performance in primary school environments. First, as children transition from primary to secondary school, their curricular experiences become more differentiated. As I have noted elsewhere (Ackerman 2017, forthcoming), at least in the USA, in contrast to the primary school curriculum which is largely ‘common’ to most of the student population, approximately one-third of the high-school curriculum consists of ‘elective courses’, whether they be in foreign languages, science, math, vocational training, and so on.

For post-secondary education, there are literally dozens or hundreds of different specialty areas for which a student may receive training or education. Moreover, within a single major, especially in the liberal arts and humanities, there are often only a few ‘core’ courses that a student must complete. Indeed, at the Ph.D. level in psychology, two students who have different specializations (e.g., industrial/organizational, cognitive/biological) may share only the most general common curriculum of statistics and research methods. This differentiation in educational content renders the comparison of process vs. content measures a fraught exercise, because no single content test or set of content tests would be suitable for students with different academic majors, or even for students with the same academic major. These issues play out in the comparison of general tests and specialized tests for both college/university selection and for post-graduate selection. The general tests (e.g., the SAT and GRE) have a history and foundation that stretches back to the first adult intelligence tests (e.g., see Lemann 1999), though they included subtests of both process (e.g., reasoning, analogies) and content (literature, science). Such tests, however, evolved to be ‘general’ so as to be appropriate to the widest sample of college-bound and graduate-school hopeful students. To accomplish this task, the content of these tests has been refined to only include curriculum that is *common* to the core curriculum of the high-school classroom. The most illustrative aspect of this is to examine the math content of the tests. Essentially the math content of the SAT is limited to algebra and geometry, topics that are required of nearly all academically oriented high schools, even though many college-bound students have completed courses in trigonometry and calculus. Ironically, the GRE general tests typically completed in the final years of college/university study have the same limitations in their coverage of math topics as the SAT, a consequence of the fact that even though some science, technology, and engineering students progress far beyond algebra and geometry courses, many college/university students take little or no advanced math courses beyond those completed in high school.

Therefore, it is particularly difficult to construct and administer content intelligence tests that are particularly suitable to large numbers of late adolescent and young adult samples. Instead, one is left with either testing, as Cattell (1957) suggested, process measures and ‘historical Gc’ tests, which leave out a substantial amount of intellectual content beyond what was learned in high school. The only extant partial solution to this problem is to administer one or more specific ‘content’ tests that are better suited to students who have completed courses in high school (e.g., the SAT II tests or the Advanced Placement Tests) or particular courses in college/university study (e.g., the GRE Subject tests). Such tests tend to predict post-secondary and graduate grades at a level equivalent to, or better than, the general tests (e.g., see Ackerman et al. 2013; Sadler and Sonnert 2010). While some researchers might reasonably assert that such mainly content measures are a close approximation to Cattell’s concept of ‘current Gc’, other researchers would classify these measures as ‘achievement’ tests, reflecting a vexing problem of distinguishing between intelligence and achievement constructs.

Nonetheless, when one considers that each formal course completed by a student prior to university study or set of courses within a domain might represent the differentiation of their intelligence into new abilities not otherwise included in most CHC conceptualizations, the number of content abilities that might underlie adult intelligence becomes very large indeed. One consequence is the breakdown of a single omnibus assessment of intelligence for adults that adequately samples content abilities (see Ackerman forthcoming, for further discussion). This conundrum does not appear to concern those researchers and theorists who advocate for exclusive testing of the process components of intelligence, and the dismissal of the role of content abilities when assessing adult intelligence. In the search for the ‘fundamental’ underlying process determinants of intelligence, researchers have largely ignored the possibility that different processes may be involved for the intelligence of children and adults.

## 15. Beyond Process and Content in Intelligence Assessment

As mentioned earlier, debates about the breadth of the intelligence construct, and the number of separable abilities to assess with an omnibus intelligence test have waxed and waned over the past 100+ years. At one end of the spectrum are those who seek a single underlying ability representing intelligence, whether it be Spearman’s *g*, working memory, or some other construct. At the other end of the spectrum, Guilford (1988) claimed the existence of at least 180 different intellectual abilities. Most intelligence assessments used beyond laboratory research have taken a middle-ground approach that largely represents Binet’s original suggestions—a relatively modest number of ability measures that can be aggregated to provide an overall estimate of an individual’s general level of intellectual ability. Even intelligence tests that follow the CHC framework typically focus on the assessment of a dozen or fewer higher-level abilities, even though there is research evidence for far more identifiable intellectual abilities (e.g., see Carroll 1993; Horn 1989).

More recent proposals for both the theory and content of intellectual abilities have been offered that do not merely represent a restatement of the Spearman or CHC approaches (e.g., see Schneider and McGrew 2018, 2019; Kovacs and Conway 2016, though see Ackerman 2016 for a critical view). It is difficult not to be skeptical whether these attempts to refine the process orientation move the field in a positive direction, especially when so little, if any, attention is paid to the correspondence between the theoretical model and prediction of individual differences in learning and performance in academic settings.

Because the CHC framework, like most other well-known hierarchical theories and models of intelligence, are ‘open’, that is, they allow for the addition of additional abilities, it is not surprising that as additional data accumulate, other replicable abilities are added to the model. This is all well and good from a theoretical perspective, but it remains to be seen whether these additional ability inclusions provide incremental predictive validity for real-world criterion predictions.

From a practical perspective, test developers realize that schools and psychometricians will typically only have a limited amount of time to administer an intelligence assessment, something on the order of a few hours. As such, they must confront the bandwidth-fidelity dilemma; that is, with a limited amount of administration time, they can assess high fidelity with narrow bandwidth (i.e., a limited number of abilities), or high-bandwidth (a broad assessment of intelligence) with lower fidelity (i.e., the lack of precision in assessing individual abilities). Matching the bandwidth of the assessment to the breadth of the criterion, as suggested by Wittmann and Süß (1999), is expected to maximize the potential for criterion-related validity. In this framework, one generally assumes that a broad assessment of school grades would be best predicted by a high- bandwidth, but a relatively low fidelity assessment of intelligence. Thus, even though theorists and researchers have identified hundreds of different intellectual abilities, it is unlikely that, say, beyond a half-dozen composites, the expansion to precise assessments of lower-order abilities is likely to be either practically feasible or result in substantial increments to predictive criterion-related validity for school grades.

## 16. Conclusions

The main sense of the literature on process and content sources of intelligence as predictors of academic success is that far too little documented evidence is available to reach definitive conclusions, even after well over 100 years of research and practice. Even though some extant IQ-type assessments have evolved to include measures of various process measures that are ‘current’ to the theories from experimental psychology (most notably, working memory), they have failed to examine whether such measures have any incremental validity for predicting academic success. One might attribute this as an indictment of the laziness of test developers, who have substituted numerous less intensive sources of validation for their measures in the past century—focusing on the correlations between their intelligence measures and other extant intelligence measures or standardized achievement tests for validation evidence rather than direct assessments of academic success. It is also an indictment of those who have advocated for ‘pure’ measures of intelligence or Spearman’s *g*, with no investment of the time and effort necessary to determine whether such measures have any validity for real-world behaviors (e.g., see Vernon and Parry 1949); leaving their validations to correlations with measures that have little or no useful validity for academic performance or any other indicators of intellectual functioning, such as the Raven’s Progressive Matrices or Cattell’s Culture-Fair Test. Fundamentally, the potential error in the reasoning of these efforts is ignoring the very real likelihood that there is much less overlap between some new process measure of intelligence and academic performance than there is between the new measure and a test like Raven’s or Cattell’s—which are mainly other process measures, not robust measures of both content and process that appear integral to the individual differences in academic success.

The existing data, such as they are, appear to suggest that when it comes to predicting individual differences in academic success, content measures show relatively higher validity than process measures, and these differences become more pronounced for older adolescents and young adults, compared to the assessments of children in early school grades. Whether this conclusion partially *explains* why there are no notable commercial process-dominated intelligence tests that have higher validity for predicting academic success than the mixed content and process measures, or content-dominated measures, or there are other reasons for the failure of process advocates to develop and validate their measures, remains to be seen. Of course, there is an entirely different discussion for contrasting process and content measures for clinical diagnosis, instead of predicting academic success, but that is a story left for another occasion. At this point in history, it is clear that the approach advocated by Spearman and his followers has not provided any justification to displace Binet–Simon and their followers in how to assess intelligence for predicting academic success.

## Data Availability

Not Applicable.
